# Toward a unified understanding of climate anxiety: Examining measures of climate and eco-anxiety

**DOI:** 10.1371/journal.pone.0339562

**Published:** 2025-12-29

**Authors:** Lisa Marie Hempel, Mattis Geiger, Cornelia Betsch

**Affiliations:** 1 Implementation Science, Bernhard Nocht Institute for Tropical Medicine, Hamburg, Germany; 2 Institute for Planetary Health Behavior, University of Erfurt, Erfurt, Germany; Wyższa Szkoła Kształcenia Zawodowego: University College of Professional Education, POLAND

## Abstract

Despite recent research efforts to understand climate anxiety, related measurement is challenged by multiple definitions and the emergence of several seemingly similar measures, warranting a thorough and joint investigation of their validity. This study (N = 1003) examined the factor structure of existing measures, namely the Climate Anxiety Scale (CAS) and the Hogg Eco-Anxiety Scale (HEAS), and tested their overlap in joint measurement models. The results showed that CAS and HEAS indeed seem to capture the same overarching construct, climate anxiety. A joint measurement model with a single higher-order general factor indicated by a CAS g-factor and four HEAS factors had good fit. This overarching climate anxiety factor was related to but still distinct from nomologically related constructs (e.g., climate-related risk perception, neuroticism). Climate anxiety was also strongly correlated with political participation, an aspect of the readiness for climate action. A small correlation with self-efficacy to show individual emission-reducing behavior and no association with climate-change-related fatigue suggests that high levels of climate anxiety are not accompanied by paralysis but instead by mild activation. The results showed generally low levels of climate anxiety in the general population. These findings are discussed regarding the resulting challenges for validation efforts and experimental work, accompanied with recommendations for further steps in measurement development.

## Introduction

Given the existential threat of climate change [1], there is a growing body of research on emotional responses to climate change, mostly regarding climate anxiety [2]. As defined by the Climate Psychology Alliance, climate anxiety refers to “heightened emotional, mental or somatic distress in response to dangerous changes in the climate system” [3, p.25]. Climate anxiety can arise from direct experiences of climate-related harm as well as from the perception and awareness of climate change and its consequences [4]. A meta-analysis has shown that certain sociodemographic characteristics, such as younger age or female gender, are more strongly associated with climate anxiety [5]. However, sociodemographic factors show weaker associations with climate anxiety compared to factors such as perceived direct exposure to climate change consequences, indirect exposure to climate change-related information, pro-environmental norms, and neuroticism [5]. Climate anxiety, just as any anxiety, can become clinically significant, affecting the ability to sleep, work, and socialize [6]. However, the literature emphasizes its adaptive functions and, in particular, warns against pathologizing climate anxiety since it is a response to a real and urgent threat [4,7–9].

A key scientific challenge lies in the diverse definitions of this phenomenon. The terms climate and eco-anxiety themselves are used across multiple discourses, including as an everyday emotion related to worry and fear, a valuable emotional response to threats like climate change that may motivate climate action, as a psychodynamic concept linked to underlying conflicts, as an existential state, or as pathological condition [10,11]. Another aspect of this conceptual ambiguity concerns the temporal nature of these constructs, as existing measures do not clearly specify whether they capture state-like fluctuations or more stable, trait-like tendencies, although certain instruction anchors may hint at one or the other conceptualization. This definitional diversity contributes to the tension in measurement approaches: between capturing severe, life-impairing mental health consequences and a natural affective response that may facilitate the necessary climate action. This may have led to the paradox in current research that although measurement approaches have focused on mental health impairment, validation studies have so far been conducted exclusively in general population samples. Such samples typically show low levels of clinically impairing symptoms, resulting in restricted variance in symptom-related items and therefore potentially underestimating associations.

Another challenge is the emergence of several seemingly similar measures within this field that claim to capture two distinct concepts. In addition to climate anxiety, eco-anxiety has been described as a similar construct with a broader context that extends beyond climate change to include anxiety related to other environmental crises, such as species extinction and deforestation [12]. Although environmental psychology has historically examined a wider range of environmental issues, research on the social and psychological impacts of climate change has expanded rapidly in recent years. However, it is unclear whether climate anxiety and eco-anxiety are perceived as distinct concepts by individuals or whether people struggle to differentiate between them. If respondents do not conceptually distinguish them, this would be reflected empirically in the very high correlations between the constructs and a joint measurement model outperforming separate measurement models. This would constitute a jangle fallacy, in which two labels are used for what is essentially the same construct [13]. Jangle fallacies pose several problems for scientific progress: they fragment the literature, obscure construct boundaries, hinder cumulative theory building, and make it difficult to compare findings across studies because researchers may believe they are studying distinct phenomena when they are not [14,15]. Clarifying whether climate anxiety and eco-anxiety are truly distinct is therefore essential.

For both climate and eco-anxiety, there is one dominant scale each. Interestingly, both two scales are used in research examining the psychological impacts of climate change (e.g., in this meta-analysis by Kühne and colleagues, [5]). A recent review by Jarrett and colleagues [16] identified several other measures with similar item content or construct names, such as the Climate Change Worry Scale [CCWS; 17], the Environmental Worry Questionnaire [EWQ; 18], or the Climate Change Distress and Impairment Scale [CC-DIS;^19^], among others. However, Jarrett and colleagues [16] also highlight that the Climate Anxiety Scale [CAS; 6] and the Hogg Eco-Anxiety Scale [HEAS; 12] are the most frequently used measures in the field, which is why we focus on these two scales in this work.

The Climate Anxiety Scale (CAS) was originally developed by Clayton and Karazsia [6] to capture a “clinically significant anxious response to climate change” (p. 9). While the initial development included a broader set of items and factors, the final CAS comprises two factors: cognitive–emotional impairment and functional impairment. However, the German version [9] identified a different two-factor structure—behavioral symptoms and cognitive consequences—excluding one item. These two factors differ from those found in Clayton and Karazsia’s [6] original validation. Since the original factor structure has been difficult to replicate in many studies [for an overview, see 20], there is ongoing debate about the CAS measurement model. Furthermore, contradicting the existing debate on measurement models, some studies have used the CAS as a unidimensional construct, justified by the high correlation of the factors [21–23]. The CAS has also been criticized for not capturing the emotional core of climate anxiety, which some argue distinguishes it from other emotions [9].

The Hogg Eco-Anxiety Scale (HEAS; [12]) was developed to measure the broader construct of eco-anxiety, encompassing anxiety related not only to climate change but also to other environmental crises. However, this broader context is primarily reflected in the instructions of the HEAS, which ask respondents how often they have been bothered by the following problems over the last two weeks when thinking about climate change and other environmental concerns, such as species extinctions, ozone hole or ocean pollution. The actual items of the scale, however, rarely mention climate change or other environmental concerns and largely focus on possible impairments across four factors: i) affective symptoms (aiming at overcoming a CAS shortcoming), ii) rumination, iii) behavioral symptoms, and iv) anxiety about one’s negative impact on the planet. While the four-factor structure of the HEAS has been successfully replicated [20], the high correlations between factors have led some researchers to adopt a more parsimonious single-factor model in practice [24,25].

Studies evaluating both scales together revealed a significant overlap, evident from high correlations between the scales (r = .58–.69, depending on the subscales;[20]). However, these correlations have been examined only at the manifest level (i.e., using aggregated scale scores). Given that measurement error attenuates correlations between aggregated scores [26] and considering the imperfect internal consistency of the scales (e.g., as reflected in their Cronbach’s alphas), the true correlation between climate anxiety and eco-anxiety is likely even higher. Thus, to assess the constructs’ distinctiveness, disattenuating approaches, such as latent variables from confirmatory factor analyses or structural equation modeling, should be used. These approaches account for measurement errors and allow researchers to examine how much variance is shared between constructs, regardless of differences in their operationalization. Applying these methods can clarify whether the constructs measured by the CAS and HEAS are truly distinct or whether they largely capture the same overarching phenomenon, warranting reconsideration of separate scales.

While both the CAS and HEAS have been subject to some validation efforts, broader validation within a nomological network of related traits remains limited. Both scales assess psychological responses to environmental concerns but differ in scope and focus. The CAS primarily measures cognitive and functional impairments associated with climate change–related distress, while the HEAS captures a broad range of responses, including rumination, behavioral symptoms, and anxiety about one’s environmental impact. Although both scales assess aspects of distress comparable to clinically relevant psychological symptoms, their validity has been evaluated mostly by correlating them with self-rated symptoms of depression and anxiety. These studies were conducted in general populations and non-clinical samples, which typically exhibit low symptom levels and, thus, skewed distributions. This may partly explain why these studies revealed small to moderate relations between climate anxiety and symptoms of depression and anxiety [9,12,20,21,24,27–29]. Moreover, the selection of such validation constructs, particularly when they are not clearly labeled to establish discriminant validity, suggests a tendency to pathologize climate and eco-anxiety. However, climate anxiety is a reaction to the existential threat of climate change and may therefore represent an adaptive reaction that can promote pro-environmental behavior [9]. Given this, neuroticism may represent a more fitting nomologically related covariate for establishing discriminant validity in the general population. Neuroticism, as a stable personality trait, applies across general and clinical populations, and the self-reported measures of depression and anxiety are mostly redundant to neuroticism (*r* = .71 in [30]; *r* = .65–.80 in [31]). Moreover, neuroticism refers to a broad, higher-order personality trait that captures the dimensional opposite of emotional stability and is characterized by a general tendency to experience negative affect, heightened emotional reactivity, and difficulties with stress regulation [32]. As a stable disposition, it shapes how individuals respond to a wide range of environmental and internal stressors. Importantly, such broad personality traits can influence the frequency and intensity of domain-specific emotional reactions [33]. Thus, examining associations between neuroticism and climate- and eco-anxiety does not suggest conceptual overlap but helps clarify whether climate-specific anxieties merely reflect general negative affectivity or constitute a distinct set of concerns tied to climate and environmental threat. Consequently, assessing the correlation between climate anxiety, eco-anxiety, and neuroticism in the general population is an important next step in the validation endeavor.

Beyond constructs related to the neuroticism dimension, other covariates are essential for understanding climate anxiety. Currently, climate and eco-anxiety are primarily defined based on the item content of the existing scales [6,12] and still lack a clear theoretical definition [10]. This lack of conceptual clarity is problematic, as climate anxiety is a broad term that is used differently across various discourses; it can refer to an emotion akin to fear and worry, strong psychological symptoms, or a phenomenon described by existential or psychodynamic psychologists [11]. One way to reduce this ambiguity is to examine its overlap with other relevant constructs, such as climate change–related risk perception.

Eco-anxiety has been suggested to heighten sensitivity to environmental challenges and encourage cognitive engagement with them [10]. Supporting this idea, climate and eco-anxiety show a substantial correlation with climate change–related risk perception [20]. Additionally, it has been debated whether these forms of anxiety function as practical anxiety [10], potentially motivating climate action. Perceived self-efficacy may play a crucial role in determining whether climate and eco-anxiety foster engagement or lead to disengagement. However, some argue that climate and eco-anxiety may induce a paralyzing fear response [34], possibly contributing to climate change–related fatigue. Such fatigue would typically mean a state of weariness, exhaustion, or decreased motivation associated with issues or discussions related to climate change. It could manifest as behavioral fatigue (a reduced willingness to take action) or informational fatigue (feeling overwhelmed or disinterested in hearing more about the issue; cf. pandemic fatigue [35,36]). To better understand the nature of climate and eco-anxiety and their functional covariates, we examined their association with risk perception of climate change–related health risks, self-efficacy to show individual emission-reducing behaviors, and climate-related fatigue.

Finally, all validation efforts require criterion validation. Climate and eco-anxiety are hypothesized to explain individual differences in climate mitigation behaviors. Earlier work found small to moderate correlations with pro-environmental behavior (CAS [6,29]; HEAS [37]; another eco-anxiety measure [38]). However, these studies often assessed a limited range of behaviors, frequently omitting high-impact actions. For example, while household waste recycling has been examined [29], behaviors with a significantly larger effect on individual CO_2_ emissions have been overlooked, such as heated home square footage per person or electricity consumption for air conditioning, depending on typical temperature differences across locations. Thus, correlations with high-impact behaviors will shed light on the question of whether greater climate or eco-anxiety is related to more relevant pro-environmental behavior. Beyond individual behavior, other relevant criteria include acceptance of environmental policies and willingness to engage in political participation. Expanding the focus beyond personal behavior change is crucial, as system-level transformations are essential for addressing climate change [39,40]. A recent development in this field is the readiness for climate action model [41,42], which combines individual behaviors weighted according to CO_2_ impact, with these system-level behaviors—that is, acceptance of climate policies and political participation—as indicators.

In sum, different measures exist that assess climate change- and eco-related anxiety; the degree of their overlap is not yet clear, and validation efforts require the use of relevant convergent constructs and criteria that are appropriate for general population, non-clinical samples. The present study therefore aims at investigating the validity of climate and eco-anxiety by testing competing measurement models for the CAS and HEAS scales that are frequently discussed in the literature [6,9,12] as well as joint models for both scales. We then test the correlation of the emerging factor(s) with neuroticism, risk perception of climate change–related health risks, climate change–related fatigue, and self-efficacy to show individual emission-reducing behavior. Moreover, we investigate the emerging factors’ criterion validity as correlations with the indicators of readiness for climate action.

## Materials and methods

### Data collection and participants

Data for this study was collected as part of a larger project, the Planetary Health Action Survey (PACE; [42,43]). PACE is a serial cross-sectional online survey, with each wave consisting of a non-probabilistic sample of approximately 1000 participants. The sample is quota-representative of Germany’s adult general population (18–74 years) for the characteristics of federal state (non-crossed) and age and gender (crossed, meaning quotas set for each age–gender combination). For the sample characteristics, see S1 Table in the Open Science Framework (OSF; https://osf.io/wvbd3/). We used data from the September 2023 PACE wave collected September 19–20 through an online access panel provider (Bilendi). Because the panel provider maintains reliable population quotas only for individuals aged 18–74, participants were drawn from this age range, and only complete responses were included in the final dataset (N = 1003). The study was hosted on the platform Tivian/Unipark. Participants needed, on average, 27 minutes to complete the full survey.

With the given sample size, one-sided testing, a significance threshold of ɑ = .05, and a power of 1 – β = .95, our sensitivity allows for the detection of a true correlation of ρ = .10 [44].

### Ethical approval

The PACE study received ethical approval from the Institutional Review Board at the University of Erfurt (No. #20220525) and is in line with the general ethical guidelines of the University of Erfurt and the German Research Foundation. Participants were informed about confidentiality, anonymity, and data-sharing practices, and they provided their informed consent prior to enrollment in the online study. In addition, data collection and analysis were conducted anonymously.

### Measures

The measures are presented in the order used in the study. The German and English versions of all measures from the PACE study, as well as the data and code for calculating the variables and reliabilities, can be found in the OSF (https://osf.io/wvbd3/). Due to copyright, please refer to the original works for the German and English versions of the CAS, HEAS, and neuroticism items (CAS [6,9]; HEAS [7,12]; Neuroticism [45]). Since this study was part of a larger data collection, more variables were assessed than are reported here. More details are reported in the project’s study protocol [42].

Constructs were assessed in blocks, with all items within each block presented in randomized order. All variables were modeled as latent constructs (for details on the models for risk perception of climate change–related health risks, self-efficacy to show individual emission-reducing behavior, and readiness for climate action, see [41]). Reliability was estimated as factor saturation McDonald’s ⍵, since it provides a more appropriate and robust measure of reliability than more common indices (e.g., Cronbach’s alpha [46]). For climate and eco-anxiety, reliability is reported for the best measurement model among the tested competing models.

#### Risk perception of climate change–related health risks.

Risk perception of climate change–related health risks was assessed using 18 items [41,42]. Participants rated nine specific health risks linked to climate change (e.g., extreme weather events and increasing heat and heat waves) on two dimensions: perceived probability (“Please indicate in each case how likely these consequences of climate change are to occur in your lifetime“; 1 = *highly unlikely* to 7 = *highly likely*) and perceived severity (“Now please indicate in each case how dangerous you think these consequences of climate change are for your life“; 1 = *harmless* to 7 = *dangerous*).

Since probability and severity ratings of the same health risks were highly correlated (despite other theoretical conceptualizations [47]), we modeled nine parcels by calculating the means of the two variables per health risk. Reliability for this model was high, with factor saturation ⍵ = .914 [46].

#### Climate change–related fatigue.

To capture behavioral and informational fatigue, we used the following two items: “I am losing my drive to fight climate change” and “I am tired of hearing about climate change.” In the Pandemic Fatigue Scale (PFS [35]), from which the items originate, the two items loaded highest on the factors of behavioral and informational fatigue and were adapted to the context of climate change. Answers were rated on a seven-point Likert-type scale ranging from 1 (*strongly disagree*) to 7 (*strongly agree*). In our study, both items, loadings constrained to equality, were modeled as one factor, showing a reliability of ⍵ = .652.

#### Self-efficacy to show individual emission-reducing behavior.

Self-efficacy regarding individual climate protection behaviors was measured using four items assessing the difficulty or ease of showing high-impact individual behaviors that reduce emissions (e.g., “Refraining from private air travel”; see [42]; one item per sector of mobility, housing and energy, food, and other consumption behavior). Participants rated the perceived difficulty of showing each behavior on a seven-point Likert-type scale ranging from 1 (*extremely difficult*) to 7 (*extremely easy*). The four items were modeled as one factor and showed a reliability of ⍵ = .564 in our study.

#### Readiness for climate action.

The readiness for climate action is defined as a latent trait indicated by individual high-emission-related behavior, acceptance of climate change mitigation policies, and political participation [41]. The three indicators are measured as follows.

Individual behavior was operationalized using the 21 behaviors most impactful in terms of individual CO_2_ emissions in Germany and was rated on impact-based response scales. Behaviors were sampled from the domains of mobility, housing and energy, food, and other consumption behavior (e.g., food: “I buy organic food”). Most answers were collected as behavioral frequency ratings ranging from 1 (*never*) to 7 (*always*), but there were some exceptions—for example, for annual distance driven by car (collected as kilometers), room temperature in winter (in degrees Celsius), duration of a shower (in minutes), and type of energy supply. To calculate the total score [42], items are weighted and thus approximate the actual footprint as measured using a CO_2_ calculator [48].

Policy acceptance was measured using 12 items asking participants about their agreement with policies covering guiding principles and policies pertaining to the mobility, housing, energy, and food sectors (e.g., food: “Subsidies in agriculture should only be available for climate-friendly farms”). Germany’s Citizen Climate Council recommendations constitute the basis for the policies (for details on the selection process, see [42]). Answers were rated on a scale from 1 (*strongly disagree*) to 7 (*strongly agree*).

Political participation was operationalized using 11 items [42] measuring conventional participation, activism, and peer group–related participation behavior (e.g., conventional: “I sign petitions for more climate protection”) on a scale ranging from 1 (*never*) to 7 (*always*). Items were adapted from several existing scales measuring political participation and contextualized to the topic of climate change [42].

In sum, the readiness for climate action is modeled in a bifactor model with a general readiness factor and specific (orthogonal) factors for each indicator group (individual behavior, policy acceptance, and political participation [41]). In the present sample, the readiness factor was reliable, with ⍵ = .708. The specific factors had acceptable reliability for political participation (⍵ = .637) and low reliabilities for individual behavior (⍵ = .176) and policy acceptance (⍵ = .251). Given that specific factors in a bifactor model are orthogonal to all other factors and that shared variance is explained by the general readiness factor, this lower reliability is to be expected [41]. In addition, in latent level analyses low reliabilities do not limit the interpretability of factors.

#### Climate anxiety.

To measure climate anxiety, we used the German version of the Climate Anxiety Scale (CAS; [9]). After reading the instruction “Below you can see some statements about the emotional consequences of climate change. Please indicate to what extent the following statements apply to you personally.” participants rated their levels of impairment across various climate anxiety symptoms. For example, items from the German factor structure included *behavioral symptoms* (“I find myself crying because of climate change”) and *cognitive consequences* (“I think, ‘why can’t I handle climate change better?’”). Responses were provided on a seven-point Likert-type scale ranging from 1 (*strongly disagree*) to 7 (*strongly agree*). Notably, the German response scale used differs from the five-point Likert-type scale used in Clayton and Karazsia’s [6] original version.

As discussed above, factor structure varies across languages and studies. Therefore, we will test competing measurement models based on the literature. The reliability of the best-fitting model of climate anxiety in this study, a g-factor model, was high (⍵ = .958).

#### Eco-anxiety.

The German version of the Hogg Eco-Anxiety Scale (HEAS [7]) was used to measure eco-anxiety. The instruction of the scale reads: “Over the last 2 weeks, how often have you been bothered by the following problems, when thinking about climate change and other global environmental conditions (e.g., global warming, ecological degradation, resource depletion, species extinction, ozone hole, pollution of the oceans, deforestation)?“

The scale consists of 13 items covering four symptom dimensions: *affective symptoms* (“Feeling nervous, anxious or on edge”), *rumination* (“Unable to stop thinking about future climate change and other global environmental problems”), *behavioral symptoms* (“Difficulty sleeping”), and *anxiety about personal impact* (“Feeling anxious about the impact of your personal behaviors on the earth”). Participants rated the frequency of these experiences over the past two weeks on a four-point scale (0 = *not at all,* 1 = *several of the days,* 2 = *over half the days,* 3 = *nearly every day*). The final higher-order measurement model for eco-anxiety with four first-order factors showed high reliability of the second-order factor, with ⍵ = .940.

#### Neuroticism.

Neuroticism was measured using nine items from the Trait-Self Description Inventory (TSDI [49]). These items, a subset of neuroticism items from the TSDI, were heuristically selected to optimize construct validity [45]. Participants responded on a seven-point Likert-type scale ranging from 1 (*strongly agree*) to 7 (*strongly disagree*)*.* An example item is “I’m often fearful that I will fail to reach my goals.”

Due to extremely high facet loadings (up to *λ* = .826) on a general neuroticism factor, deviating from Olaru et al. [45], we modeled only a general factor of neuroticism indicated directly by the nine items—a model also used in other studies [e.g., 50]. This factor demonstrated high reliability (⍵ = .912).

### Statistical analysis

For all constructs used in this manuscript, we tested measurement models and evaluated their fit. Model fit was deemed good with a comparative fit index (CFI) and Tucker–Lewis index (TLI) ≥.950, root mean square error of approximation (RMSEA) <.050, and standardized root mean square residual (SRMR) <.080, and acceptable with CFI and TLI ≥ .90, RMSEA < .08, and SRMR < .11 [51–53].

We first tested and compared competing measurement models for each construct separately. After identifying the best-fitting and most parsimonious model for the CAS and HEAS, we tested their overlap in joint measurement models. Given that our goal was to assess whether both scales capture the same overarching construct, we did not exclude potentially redundant items that measured similar content across the two scales beforehand.

For covariates—including risk perception of climate change–related health risks, self-efficacy to show individual emission-reducing behavior, readiness for climate action, and neuroticism—we used established models from the literature. All correlations between constructs were estimated at the latent factor level, meaning that they were disattenuated for measurement error [26]. Latent factors were identified by standardizing them (i.e., fixing their variance to 1). Models were estimated using robust maximum likelihood (MLR) estimation, and statistical significance was assumed at ɑ = .05.

Analyses were conducted using R (v4.3.1; R Core Team 2023) employing the packages lavaan (v0.6.16 [54]), semTools (v0.5.6 [55]), ggridges (v0.5.4 [56]), viridis (v0.4.2 [57]), ggplot2 (v3.4.3 [58]), maditr (v0.8.3 [59]), and dplyr (v1.1.3 [60]).

## Results

First, we calculated descriptive measures for the CAS and HEAS. For both measures, items were not normally distributed, showing pronounced floor effects (https://osf.io/wvbd3/, S1 and S2 Figs).

### Measurement models of climate and eco-anxiety

**Fig 1:**
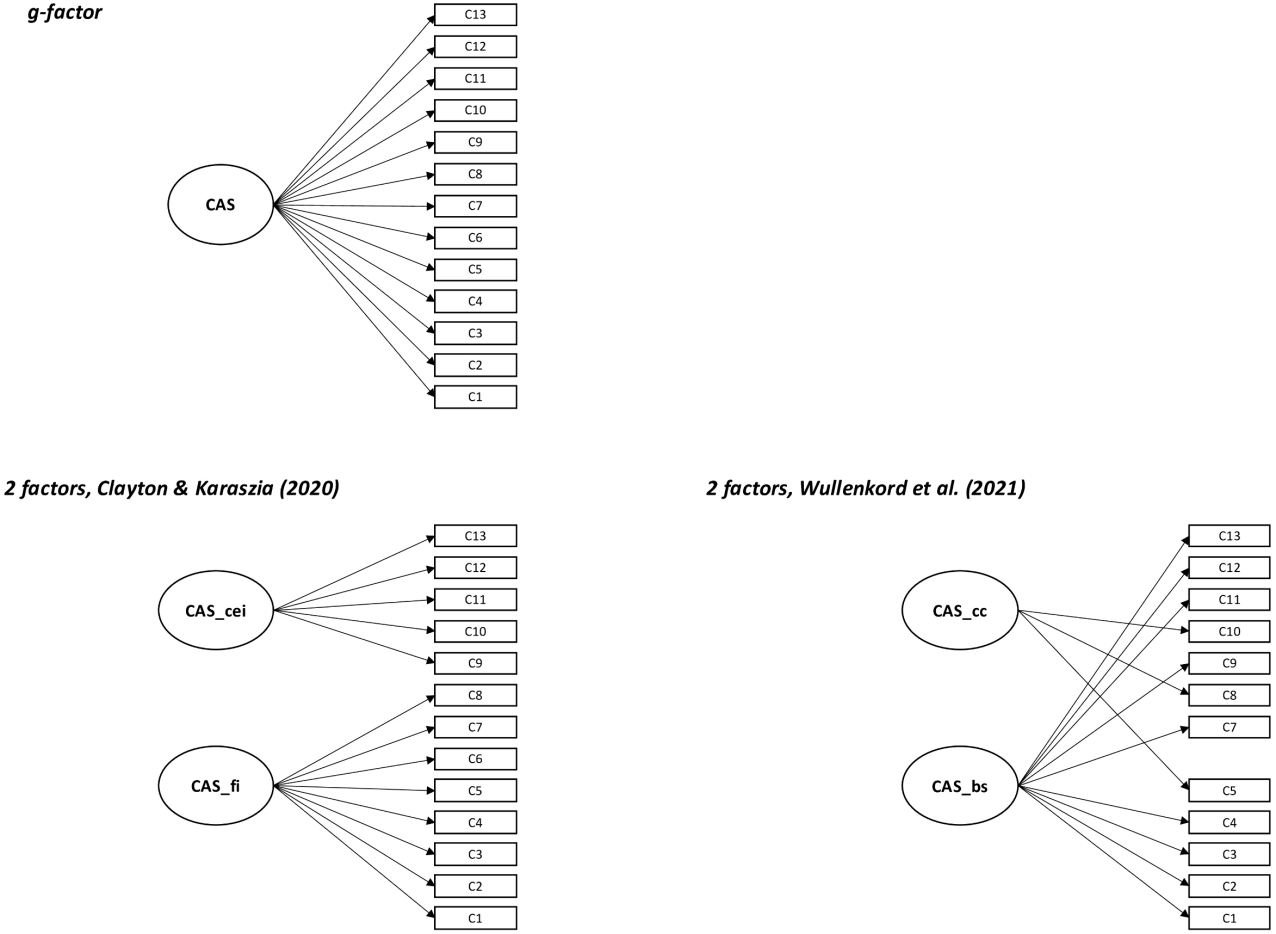
Scheme plots of all tested models for the Climate Anxiety Scale (CAS). *Note*. CAS_cei = cognitive-emotional impairment; CAS_fi = functional impairment; CAS_cc = cognitive consequences; CAS_bs = behavioral symptoms.

**Fig 2:**
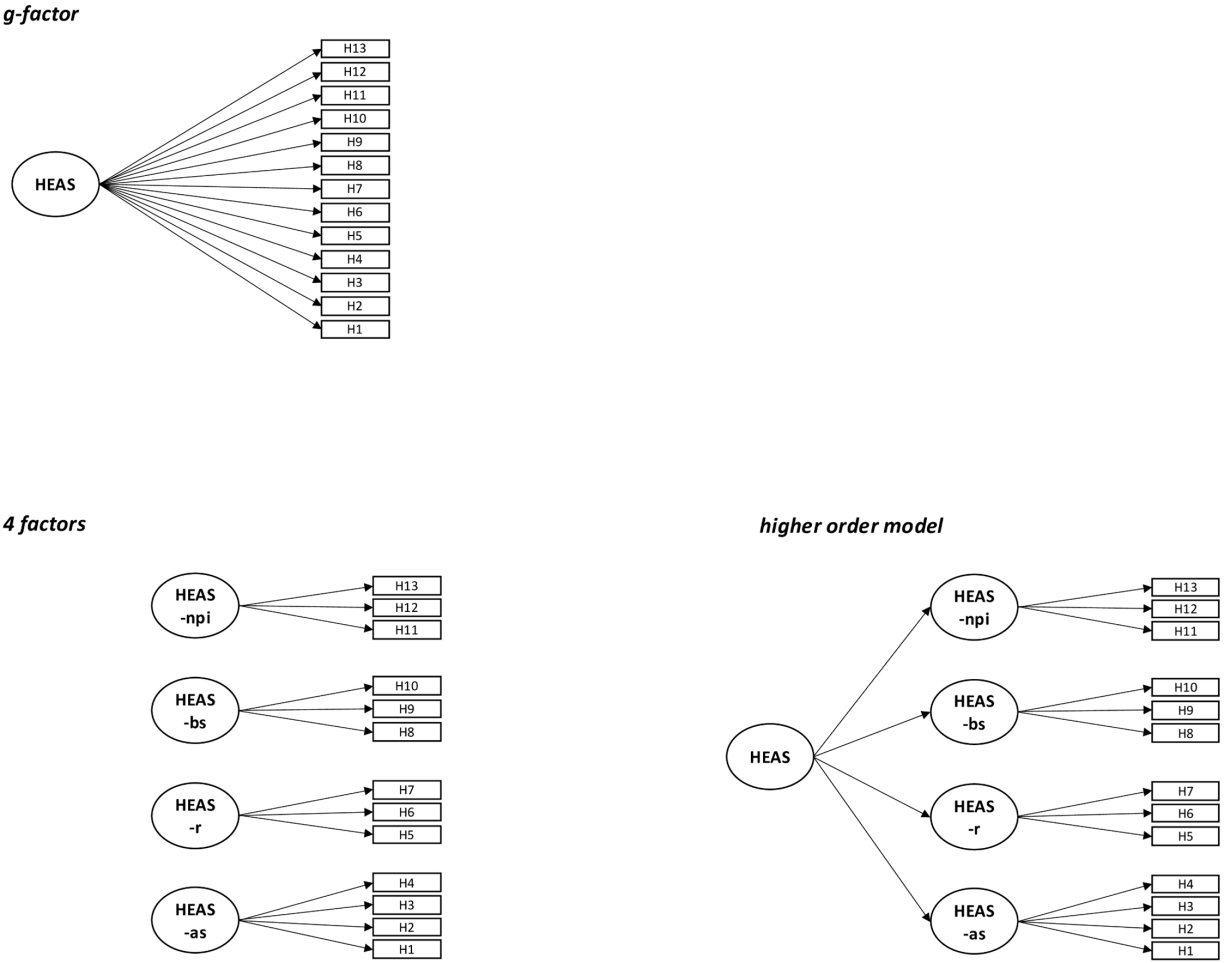
Scheme plots of all tested models for the Hogg Eco-Anxiety Scale (HEAS). *Note.* HEAS-as = affective symptoms; HEAS-r = rumination; HEAS-bs = behavioral symptoms; HEAS-npi = anxiety about negative personal impact.

**Fig 3:**
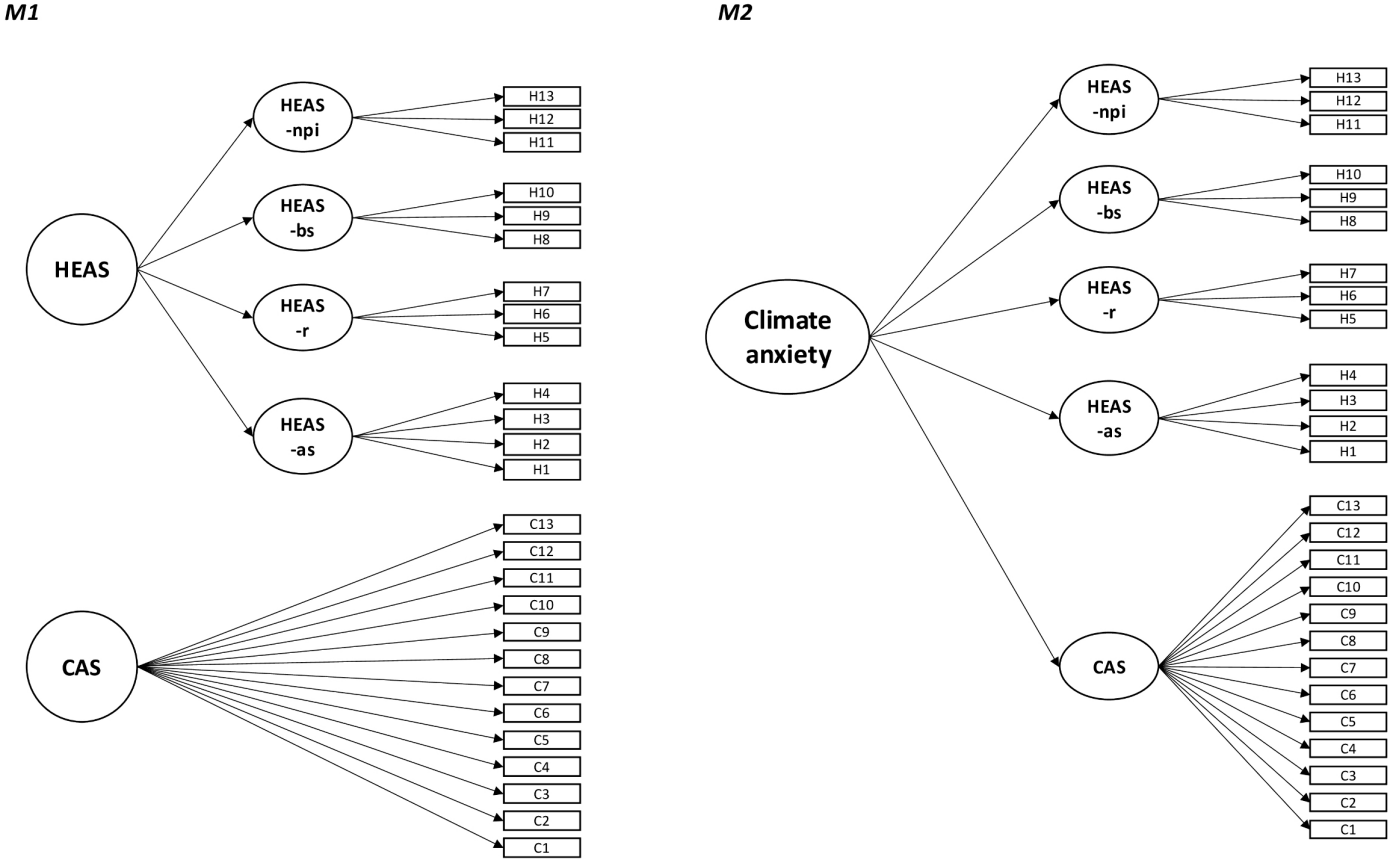
Scheme plots of the tested joint models for climate and eco-anxiety. *Note.* M1 = Model 1; M2 = Model 2; HEAS-as = affective symptoms; HEAS-r = rumination; HEAS-bs = behavioral symptoms; HEAS-npi = anxiety about negative personal impact.

Using confirmatory factor analyses, we tested several models frequently discussed in the literature for both the CAS and HEAS. The fit indices for these models are presented in Tables 1 and 2. Additionally, in Figs 1 and 2, scheme plots illustrate the tested models for CAS and HEAS, respectively, while Fig 3 presents scheme plots for joint models incorporating both measures.

**Table 1:** Overview of measurement models for the Climate Anxiety Scale (CAS).

Model	*X*^2^(df); *p*	CFI	TLI	RMSEA	SRMR	*X*^2^-difference test (df) to g-factor model; *p*
CAS: g-factor	*χ*² (65) = 538; *p* < .001	.962	.954	.054	.025	
CAS: two factors; Clayton and Karaszia (2020)	*χ*² (64) = 517; *p* < .001	.963	.955	.053	.025	*χ*² (1) = 9; p = .002^1^
CAS: two factors; Wullenkord et al. (2021)	*χ*² (53) = 401; *p* < .001	.971	.964	.050	.022	*χ*² (12) = 78; *p* < .001^1^

*Note. X*^2^-difference tests were calculated only for nested models. ^1^The *X*^2^-difference test included a correction applied to compensate for possible distortions caused by non-normal data or complex model structures, resulting in different results from the simple difference between both models’ *X*^2^.

**Table 2:** Overview of measurement models for the Hogg Eco-Anxiety Scale (HEAS).

Model	*X*^2^(df); *p*	CFI	TLI	RMSEA	SRMR	*X*^2^-difference test (df) to g-factor model; *p*
HEAS: g-factor	*χ*² (65) = 682; *p* < .001	.949	.938	.060	.035	
HEAS: higher-order model	*χ*² (61) = 286; *p *< .001	.985	.981	.033	.023	*χ*² (4) = 119; *p* < .001^1^
HEAS: four factors	*χ*² (59) = 213; *p* < .001	.992	.989	.025	.017	*χ*² (6) = 169; *p* < .001^1^

*Note. X*^2^-difference tests were calculated only for nested models. ^1^The *X*^2^-difference test included a correction applied to compensate for possible distortions caused by non-normal data or complex model structures, resulting in different results from the simple difference between both models’ *X*^2^.

#### CAS measurement model.

For the CAS, we tested a one-factor solution as a simple g-factor model, as well as two two-factor solutions proposed by Clayton and Karaszia [6] and Wullenkord and colleagues [9]. The model by Clayton and Karaszia [6] distinguishes between *cognitive-emotional impairment* (items 1–8) and *functional impairment* (items 9–13). In contrast, Wullenkord and colleagues [9] proposed a structure based on *behavioral symptoms* (items 1–4, 7, 9, 11–13) and *cognitive consequences* (items 5, 8, 10). Following Wullenkord and colleagues [9], we excluded Item 6 when testing their model.

The simplest model, the g-factor model, met the thresholds for acceptable to good model fit. Both two-factor solutions reached the same fit thresholds but showed extremely high factor correlations (*r* = .983/.930). Given that the g-factor model already demonstrated good model fit, was more parsimonious, and considering the very high factor correlations, we concluded that the g-factor model was the most appropriate for the CAS.

#### HEAS measurement model.

For the HEAS, we tested a g-factor model and a four-factor structure proposed by Hogg et al. [12]. These four factors included *affective symptoms* (items 1–4), *rumination* (items 5–7), *behavioral symptoms* (items 8–10), and *anxiety about one’s negative impact on the planet* (items 11–13).

The g-factor model demonstrated reasonable fit, but CFI, TLI, and RMSEA did not meet the threshold for good fit. In contrast, the four-factor solution met all fit thresholds for good fit. However, this model exhibited very high correlations between the four factors (*r* = .808 to .983), suggesting that a higher-order general factor might provide a similarly strong fit. To test this, we estimated a third model incorporating a higher-order factor—a second-level g-factor indicated by all four first-level factors from the four-factor model. This model met all fit thresholds for good fit. Given the high correlation between factors in the four-factor solution and the strong saturation of the higher-order factor (⍵ = .940), we concluded that the higher-order model was the most appropriate. It offers a more parsimonious structure while maintaining good model fit. Therefore, we recommend the use of the higher-order model for the Hogg Eco-Anxiety Scale.

#### Joint model of climate and eco-anxiety.

To examine the overlap between the Climate Anxiety Scale (CAS) and the Hogg Eco-Anxiety Scale (HEAS), we estimated joint measurement models incorporating all items from both scales.

The first joint model combined the final measurement models of the CAS (g-factor) and the HEAS (higher order), allowing both general factors to correlate (M1). This model showed good fit (*χ²* (294) = 1294, *p* < .001, CFI = .965, TLI = .962, RMSEA = .038, SRMR = .027), with a high correlation between the two factors (*r* = .831). Given this strong correlation, we tested a second joint model (M2): a higher-order structure in which a single overarching general factor was indicated by the CAS factor and the four HEAS factors. The conceptual model is shown in Fig 3, while the full model, including all factor loadings, is available in the supplementary material (https://osf.io/wvbd3/; S3 Fig). This model produced identical fit indices to the first model. Loadings were very homogeneous and large across the factors from both scales (*λ* = .831–.984), with the CAS loading being descriptively the smallest loading but still comparable to the HEAS-npi loading (*λ* = .878).

Considering the homogeneity of factor loadings in the second model, we concluded that the CAS factor fits well under the higher-order factor. Additionally, working with one global factor across both scales offered a more parsimonious approach than considering the scales separately. Therefore, we adopted the second joint model (M2) for subsequent analyses. In the discussion, we explain why we refer to this overarching factor as climate anxiety.

To allow readers to compare association patterns with validation constructs across both joint modeling approaches (M1 and M2), the supplementary material presents validity analyses in which CAS and HEAS are examined as separate correlating factors (model M1; https://osf.io/wvbd3/; see Section 7 in the file 2_CA_Paper_supplement_analysis_final2, available in both HTML and RMD formats). The results show that the two higher-order factors for HEAS and CAS display redundant correlation patterns, both in magnitude and in direction, further supporting the selection of M2 for the main analyses.

### Correlates of the climate anxiety factor

**Fig 4:**
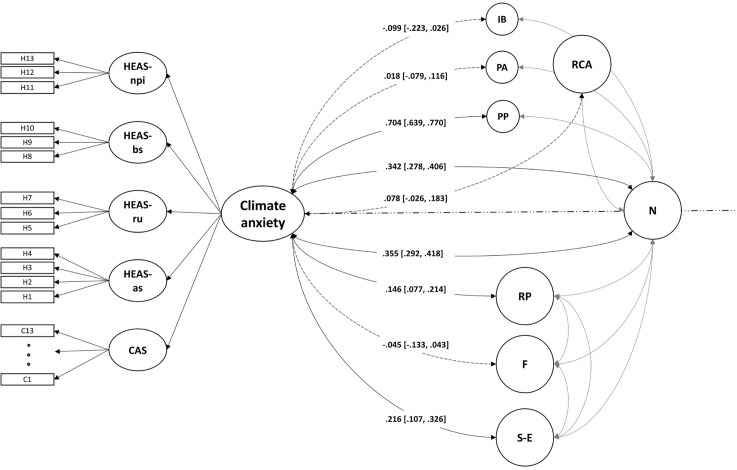
Climate anxiety model and corresponding models for construct and criterion validity. *Note*. Climate anxiety = higher-order factor of climate anxiety; CAS = CAS scale/climate anxiety; C1–C13 = Items 1–13 of the CAS scale; HEAS-as = HEAS affective symptoms; HEAS-ru = HEAS rumination; HEAS-bs = HEAS behavioral symptoms; HEAS-npi = HEAS anxiety about one’s negative impact on the planet; H1–H13 = Items 1–13 of the HEAS scale; RCA = readiness for climate action; IB = individual behavior; PA = policy acceptance; PP = political participation; N = neuroticism; RP = risk perception of climate change–related health risks; F = climate change–related fatigue; S-E = self-efficacy to show individual emission-reducing behavior. Square brackets show 95% confidence intervals. Solid lines show significant associations, with *p* < 0.001. Dashed lines show associations that are not significant. As construct and criterion validity for climate anxiety were estimated in two separate models, both including neuroticism, neuroticism is shown in the middle of both models and is correlated slightly differently to climate anxiety in the two models.

The latent correlations between the overarching climate anxiety factor and relevant constructs to test construct and criterion validity are shown in Fig 4. Models to test construct and criterion validity were calculated separately to ensure stable covariance parameters for the individual behavior factor.

In a separate model, neuroticism showed a moderate correlation with climate anxiety (*r* = .355, *p* < 0.001, 95% CI [0.292, 0.418]), indicating that the constructs are related but not redundant. Due to this non-redundancy, and for simplification, we forgo reporting relations of climate anxiety with other constructs incrementally to neuroticism in this manuscript. Nevertheless, partial correlations controlling for neuroticism are reported in the supplementary material (https://osf.io/wvbd3/; see the file 2_CA_Paper_data_analysis_final2 in both HTML and RMD formats, provided at the end of Sections 4 and 5).

Furthermore, for construct validity, we found a small correlation between the overarching climate anxiety factor and the risk perception of climate change–related health risks, while climate change–related fatigue was not associated with the climate anxiety factor. The correlation between the climate anxiety factor and self-efficacy to show individual emission-reducing behavior was also small.

For criterion validity, the climate anxiety factor was not correlated with the general readiness factor or with the specific factors of individual behavior and policy acceptance. However, we observed a strong correlation with the specific readiness factor of political participation.

## Discussion

This study examined the validity of climate and eco-anxiety by testing competing measurement models for two existing scales: the Climate Anxiety Scale [6] and the Hogg Eco-Anxiety Scale [12]. By testing joint models, we assessed the overlap between both constructs and identified a jangle fallacy. Our findings showed that the best-fitting and most parsimonious solution was an overarching model with a general climate anxiety factor, indicated by one CAS and four HEAS factors. This suggests that existing scales, such as the CAS and HEAS, seem to measure the same overarching construct, presumably climate anxiety as we discuss below. We also highlight additional limitations in the measurement of climate and eco-anxiety and propose ways to improve assessment of this important topic.

The first key finding is that most participants in this non-clinical, general adult population sample reported low levels of climate and eco-anxiety, resulting in floor effects. The fact that most people in the general population are not heavily affected by climate anxiety, meaning they do not report highly impairing anxiety accompanied by symptoms that interfere drastically with everyday life, is in line with most other studies using the scales (CAS [6,9,22,27,29] and HEAS [7,12,24,37]). Given that climate anxiety is partly defined by clinically relevant emotional and functional impairments and has been studied exclusively in general populations, these findings are not surprising. However, this limits knowledge about the validity of the construct.

The floor effect of climate and eco-anxiety in the general population is a form of variance restriction that can substantially lower correlations estimated for investigations of convergent or criterion validity. Furthermore, floor effects can introduce spurious interactions [61], which must be considered when interpreting experimental work. Since floor effects occur when scale items fail to differentiate between individuals due to uniformly low responses, a promising approach could be to distinguish between affective climate-anxious experiences (including appraisals) and their potential impairments in psychological functioning [62]. Wullenkord and colleagues [62] found that climate-anxious affect and appraisal, which generally have a higher occurrence probability, exhibit greater variability in the general population—an essential condition to avoid variance restrictions. In contrast, impairment is likely to manifest primarily in vulnerable or clinically affected groups, suggesting that future work should evaluate impairment separately and include such populations in validation efforts.

Second, our comparison of different measurement models for the Climate Anxiety Scale and the Hogg Eco-Anxiety Scale addresses the varying ways in which these scales have been used in previous research (e.g., as one-factor solutions, as separate but correlated factors, or as different factors compared to the original version). Examining the overlap between climate and eco-anxiety measures, we found that a joint higher-order model—with a single general factor—provided the best fit for the data. This suggests that treating climate and eco-anxiety as distinct constructs constitutes a jangle fallacy, as both scales appear to measure the same overarching construct, which we would describe as climate anxiety.

This interpretation may be debated. On the one hand, theoretical considerations suggest that eco-anxiety encompasses a broader construct than climate anxiety [12,63], which might support labeling the factor as eco-anxiety. However, our model reveals a substantial overlap between CAS and HEAS, and we want to emphasize the possibility that the joint model nevertheless mostly measures anxiety related to climate change. This consideration originates from closely inspecting the HEAS, which consists of several symptoms. In relation to these symptoms, other environmental threats are mentioned explicitly only in one item and in the scale’s introduction: “Over the last 2 weeks, how often have you been bothered by the following problems when thinking about climate change and other global environmental conditions (e.g., global warming, ecological degradation, resource depletion, species extinction, ozone hole, pollution of the oceans, deforestation)?” Thus, it may well be that climate change is the more dominant concept in people’s minds and that other environmental threats are overread in the introduction, so climate change–related anxiety is also more dominant in this scale. However, fully resolving this conceptual question requires additional quantitative and qualitative research to clarify which climate and/or environmental issues respondents actually think about when completing these scales, and whether they differentiate between them at all. For now, we define the overarching construct measured by both scales as climate anxiety and conclude that the CAS and HEAS items do not measure fundamentally different constructs but rather assess a single overarching construct.

The third learning from this study resulted from validity analyses of the overarching climate anxiety factor in a nomological network of related traits. The study revealed a moderate correlation between climate anxiety and neuroticism, aligning with previous research assessing the relation to depression and anxiety [9,12,20,21,24,27–29] and neuroticism [25,64]. However, the correlation was rather weak, suggesting that while neuroticism, as a stable personality trait, can predispose individuals to heightened climate anxiety, its expression may depend on the contextual activation of climate change–related information. Climate anxiety showed a limited correlation with the perception of climate-related health risks, emphasizing that these are distinct constructs with potentially varying impacts on climate action and coping behavior [65,66]. Notably, climate change–related fatigue did not correlate with climate anxiety. Self-efficacy to show individual emission-reducing behaviors displayed a small positive association with climate anxiety. In summary, our results of a small positive correlation with self-efficacy and no association with fatigue, together with low rates of climate anxiety in the general population, suggest that people in general are not currently paralyzed by climate anxiety. This contrasts with media portrayals [e.g., 67]. In theorizing, climate anxiety is attributed to both a paralyzing and a motivating effect on pro-environmental behaviors [10,11,34], and the data suggest that this relation may be more complex [66,68–70]. In sum, previous and the current analyses warrant deeper investigation of potential moderators.

Interestingly, between the overarching climate anxiety factor and readiness for climate action, we found no substantial associations, including no relations with the two specific factors of individual behavior and policy acceptance. Using other measures to assess readiness factors, previous studies have reported small associations with policy support [9] and small to moderate correlations with individual pro-environmental behavior [25,29,37,71]. The fact that we found no such associations might be attributed to the different measurement approaches used in this study, which allow for the disentanglement of the readiness for climate action in general and its orthogonal modeled indicators [41]. Moreover, we integrated only the individual behaviors that have the greatest impact in terms of individual CO_2_ emissions (e.g., living conditions) that are harder to change than low emission behaviors, which have often been included in previous studies (e.g., disposing, recycling, and waste behavior [29,71]).

Finally, we found a very strong correlation between climate anxiety and the specific political participation factor. Other studies have also reported that climate anxiety is more strongly associated with activism than with individual emission-related behavior [28,38]. However, our results do not allow for causal claims, and both potential directions of influence must be considered. On the one hand, political participation may heighten climate anxiety, as engaging in activism often involves increased exposure to discussions about climate change and its negative consequences. On the other hand, individual differences in climate anxiety may drive varying levels of political participation. A recent longitudinal study on the bidirectional relationship between climate worry and climate activism found evidence for both pathways, with a stronger effect of climate worry increasing the likelihood of political engagement in climate activism [72].

In sum, both previous studies and the current work have identified a lack of comprehensive definitions of the core constructs [10,11]; floor effects and restricted variance in the outcome variable (e.g., [6,7,12,29]); a lack of clarity in the factor structure [20]; reservations about whether the emotional core of climate anxiety is captured, especially in the Climate Anxiety Scale [9]; and finally, the insight that the two existing measures cover the same overarching construct.

To advance the field, we propose the following to improve the measurement of climate anxiety. First, it is essential to begin with clear and refined definitions of the constructs being measured, including greater conceptual explicitness regarding their temporal stability. A particularly promising approach is to distinguish between affective and appraisal reactions to climate change, on the one hand, and the resulting impairments in quality of life, on the other [62]. While the CAS and HEAS already capture aspects of impairment, further refinement of these scales could enhance sensitivity for clinical or otherwise vulnerable populations, enabling more precise assessment and validation of the consequences of climate anxiety. At the same time, this distinction will help avoid variance restrictions when measuring affective and appraisal reactions and will facilitate the development of an instrument that effectively captures the emotional basis of climate action in the general population. Additionally, such an approach will reinforce the non-pathologization of climate anxiety, a topic frequently discussed in the literature [4,7–9]. Moreover, differentiating these aspects of climate anxiety will enable more precise validation efforts, ensuring that measures of impairment undergo validation in clinical samples, while emotional experiences are appropriately studied in the general population.

To ensure replicability of future scales’ factor structures and avoid challenges related to multicollinearity, it is important to recognize that the emotional core of climate anxiety is empirically difficult to separate from other climate-related emotions. Studies have shown high correlations, especially between climate anxiety, climate anger, and climate sorrow (e.g., in the Inventory of Climate Emotions [8,73]). Given these overlaps, future research could benefit from a measure that considers a broader spectrum of emotions and their shared role in promoting climate action. By refining conceptual distinctions and overlaps and improving measurement tools, future research can provide a more comprehensive understanding of climate-related emotions and their impact on individual and collective action.

### Limitations

Some limitations of this study should be considered. Both the CAS and HEAS were developed in high-emitting countries (e.g., the US, Australia, New Zealand) with relatively advantaged populations. In many contexts where climate change impacts are already a daily reality—despite only little emissions coming from these contexts (e.g., less advantaged countries in sub-Saharan Africa or Pacific Island states)—emotional responses may differ qualitatively and quantitatively. To fully understand climate anxiety across diverse cultural and socio-economic settings, future research should test and adapt measures contextually, taking into account local experiences of climate-related impacts and vulnerabilities. Further, the study was cross-sectional and correlational, meaning that causal claims cannot be made regarding the relationships between the overarching climate anxiety factor and other constructs. The sample included only adults, limiting the generalizability of our findings to children and adolescents, although they might be a particularly relevant group for experiencing impairments due to climate anxiety [74]. Furthermore, investigating the causal relationships between climate anxiety and measures of political participation, acceptance of climate policies, and individual pro-environmental behaviors, as well as the role of self-efficacy, remains an important direction for future research. Understanding causal relations could be especially interesting in populations that rally for more climate protection. Longitudinal studies spanning longer time periods could provide deeper insights into these dynamics. However, given the identified limitations of the current measures, we recommend improving the measures before applying them in experimental or longitudinal studies.

## Conclusions

The current work addressed the complexity of measuring climate and eco-anxiety. It emphasized the challenges posed by vague definitions, partial clinical operationalizations, variance restrictions in the measures through floor effects, and proposed different measures of climate and eco-anxiety. The study provided a thorough validation of the frequently used measures of CAS and HEAS. A joint model of climate and eco-anxiety showed that CAS and HEAS presumably capture the same overarching construct: climate anxiety. Validation efforts in a nomological network of climate anxiety not only enhance our understanding of the construct but also position climate anxiety as a pivotal focus for future research. This could elucidate variance in climate activism and become increasingly relevant when climate health risks aggravate and become a greater mental health threat in the future. Before advancing future research, it is crucial to refine climate anxiety measures to prevent jangle fallacies and variance restrictions—starting with precise definitions that distinguish between emotional responses and impairments related to climate change and ensuring that refined measures are validated within their specific target groups.
